# Advances in diagnosing mild cognitive impairment and Alzheimer’s disease using ^11^C-PIB- PET/CT and common neuropsychological tests

**DOI:** 10.3389/fnins.2023.1216215

**Published:** 2023-07-10

**Authors:** Qing Zhao, Xinxin Du, Wenhong Chen, Ting Zhang, Zhuo Xu

**Affiliations:** ^1^Department of Neurology, China-Japan Union Hospital of Jilin University, Changchun, Jilin, China; ^2^Department of Sleep Medicine, Guangxi Zhuang Autonomous Region People's Hospital, Nanning, Guangxi, China; ^3^Department of Rehabilitation, China-Japan Union Hospital of Jilin University, Changchun, Jilin, China; ^4^Rehabilitation Therapeutics, School of Nursing of Jilin University, Changchun, Jilin, China

**Keywords:** Alzheimer’s disease, PET/CT, mild cognitive impairment, neuropsychological test scale, amyloid

## Abstract

Alzheimer’s disease (AD) is a critical health issue worldwide that has a negative impact on patients’ quality of life, as well as on caregivers, society, and the environment. Positron emission tomography (PET)/computed tomography (CT) and neuropsychological scales can be used to identify AD and mild cognitive impairment (MCI) early, provide a differential diagnosis, and offer early therapies to impede the course of the illness. However, there are few reports of large-scale ^11^C-PIB-PET/CT investigations that focus on the pathology of AD and MCI. Therefore, further research is needed to determine how neuropsychological test scales and PET/CT measurements of disease progression interact.

## 1. Introduction

Diagnosis and treatment of dementia is challenging and has an impact on both social care and global health. Dementia affects more than 50 million people worldwide and according to the most recent estimates, as the population ages, this number is predicted to quadruple (GBD 2016 Dementia Collaborators, 2019). According to The World Alzheimer’s Disease Report, dementia is the third most serious health issue, with enormous financial expenses, after cancer and cardiovascular disease globally (Prince et al., 2015).

Alzheimer’s disease (AD) is a neurodegenerative disease that is the leading cause of dementia and accounts for 60–80% of dementia cases (Scheltens et al., 2021). The main pathological features include the formation of senile plaques by intracerebral Aβ protein deposition and intracellular neurogenic fiber tangles by tau protein hyper-phosphorylation (Carreiras et al., 2013; Hansson, 2021; Tzioras et al., 2023). It has been shown that the prevalence of AD doubles approximately every 5 years after the age of 65 years (Hane et al., 2017). According to the latest global burden of Disease study, AD remains the fifth leading cause of death worldwide (GBD 2016 Neurology Collaborators, 2019; 2022 Alzheimer’s disease facts and…, 2022).

Mild cognitive impairment (MCI) is a transition stage between normal cognitive aging and dementia. Recent studies have shown that the prevalence of MCI in the global community exceeds 15% and that the prevalence of MCI increases with age (Bai et al., 2022). The conversion rate from MCI to AD is approximately 15% per year (Hojjati et al., 2018). Intervention and treatment of patients at the MCI stage can benefit patients by slowing the progression of the disease and reducing the conversion rate of AD.

The current clinical diagnosis of AD is usually based on the patient’s medical history, symptoms, neuropsychological test scales, blood tests, cerebrospinal fluid tests, and imaging (Hane et al., 2017; Teunissen et al., 2022). Neuropsychological scales have been widely used in clinical settings (Espinosa et al., 2017). Moreover, with the development of computer and neuroimaging technologies, it is possible to identify AD early and noninvasively. In recent years, researchers have widely used amyloid and tau imaging using positron emission tomography (PET) for the differential diagnosis of dementia, especially for the diagnosis of AD (Jack et al., 2018). Nevertheless, there are few reports on the diagnosis of AD using ^11^C-PIB-PET/computed tomography (CT) and the relevance of ^11^C-PIB-PET/CT combined with neuropsychological testing scales in AD and MCI disease progression and condition assessment. Currently, there is no established cure for dementia; however, there are treatments to impede cognitive decline or reduce dementia-related behavioral and psychiatric symptoms (Sharma, 2019). The application of PET/CT and neuropsychological examinations can improve the precision of early diagnosis of dementia and give patients the opportunity to intervene early, which is clinically important to prevent the onset of dementia and delay the progression of the disease (Oddo et al., 2004). This article reviews the progress of PIB-PET/CT with commonly used neuropsychological scales in MCI and AD.

## 2. Diagnosis of Alzheimer’s disease and mild cognitive impairment

Alzheimer’s disease is a progressive neurodegenerative disease characterized by cognitive impairment and affects activities of daily living (Rowe and Villemagne, 2011). Although the pathogenesis of AD has not been clearly elucidated, the most common mechanisms suggested include Aβ protein deposition, tau protein hyper phosphorylation, and processes involving inflammatory mechanisms, oxidative stress, mitochondrial mechanisms, and cerebrovascular mechanisms (Ferrari and Sorbi, 2021). These neuropathological changes are believed to start 15–20 years before the appearance of clinical symptoms of dementia (Trejo-Lopez et al., 2022). Their transcendental pathophysiological characteristics can be detected using cerebrospinal fluid evaluation or imaging techniques (Scheltens et al., 2021). The primary clinical symptoms include decreased cognitive function, decreased brain function, progressive memory loss, and progressive loss of self-care and psychiatric symptoms as the disease worsens, causing a great burden on the family and society (Livingston et al., 2017; Lane et al., 2018). Estimations of AD prevalence suggest that biologically defined AD is three times more prevalent than clinically defined AD (Scheltens et al., 2021). Over the past 30 years, mortality rates for AD and other dementias in China have been on the rise and have increased significantly with age (Bai and Dong, 2021). Current medications for AD only temporarily alleviate memory symptoms and other cognitive changes, but do not stop or reverse AD progression (Dong et al., 2019).

MCI, which is the prodromal stage of AD, is an intermediate stage between normal cognitive aging and overt dementia, characterized by marked cognitive decline beyond normal aging, but not meeting clinical diagnostic criteria for AD, with relative maintenance of activities of daily living (Petersen, 2004). It is believed that MCI represents a significant step in the aging process and MCI may represent an early stage of dementia with a tendency to progress to clinically diagnosed dementia. Approximately, 10–15% of patients with MCI progress to AD annually (Petersen, 2000; Gauthier et al., 2011) with a lifetime prevalence of 60–90% (Anderson, 2019). Some patients then remain stable (Diniz et al., 2008). Recent studies have shown that 12.2% of people over 55 years of age in China have MCI, and the prevalence of MCI increases with age (Lu et al., 2021).

New diagnostic criteria for AD and AD-derived MCI (Albert et al., 2011; McKhann et al., 2011) include biomarkers based on neuroimaging or cerebrospinal fluid tests that can increase the sensitivity or specificity of the diagnosis (Zhang et al., 2014). The definitive diagnosis of AD can only be made by neuropathological examination, although neuropsychological and imaging examinations are still preferred methods for clinical diagnosis of AD (Tiwari et al., 2019). The Mini Mental State Examination (MMSE) and Montreal Cognitive Assessment (MoCA) scores have substantial specificity and sensitivity in clinically diagnosing AD (Nasreddine et al., 2005; Chen et al., 2016). Physicians now widely use PET/CT examinations for early diagnosis and assessment of AD. PET/CT helps to accurately identify patients with MCI who may transition to AD or other forms of dementia.

## 3. PET/CT in Alzheimer’s disease and mild cognitive impairment

Some studies suggest that onset of AD is initiated with deposition of Aβ in the brain, followed by pathological changes in peripheral nerve cells and glial cells (Selkoe and Hardy, 2016). Aβ deposition begins before the onset of clinical symptoms, such as cognitive decline and behavioral changes (Jack et al., 2013; Zetterberg and Blennow, 2013). Identifying and quantifying amyloid deposition *in vivo* is valuable in AD research, has been used in large synergistic studies, and has value in clinical settings (Brown et al., 2019). According to the 2011 recommendations of the European and US National Aging and Alzheimer’s Societies to revise the diagnostic criteria for AD (Dubois et al., 2007, 2010; McKhann et al., 2011), the detection of Aβ deposits at the MCI stage may provide a basis for the early diagnosis of AD and timely intervention.

Molecular imaging methods are based on a visual depiction of functional or pathological changes in the brain for the clinical diagnosis of neurodegenerative diseases, and recently, amyloid and tau imaging techniques using PET have been widely used for the differential diagnosis of dementia and are important for the diagnosis of AD (Jack et al., 2018), PET imaging is to measure glucose metabolism, β-amyloid plaques, and neurogenic fiber tangles in the framework of amyloid-tau-neurodegeneration (Jack et al., 2018). Using radiotracers specific to β-amyloid plaques, PET imaging provides a useful tool for quantifying β-amyloid deposition in various brain regions.

### 3.1. Amyloid study

There is growing evidence that amyloid-PET is a valuable support for clinicians, leading to changes in diagnosis and treatment planning based on traditional workups and increasing confidence in etiologic diagnosis. Commonly used tracers for Aβ-PET include ^11^C-Pittsburgh compound B (PIB) and ^18^F labeled tracers (^18^F-Flutemetamol, ^18^F- florbetapir and ^18^F-forbetaben) (Villemagne et al., 2012; Landau et al., 2013). A multicenter study in 2019 involving 18,295 patients demonstrated that implementing PET scan led to higher diagnostic certainty (Rabinovici et al., 2019). In 2004, the first study was conducted. ^11^C-PIB-PET/CT was first performed in 2004, and ^11^C-PIB was the first PET radioactive tracer capable of *in vivo* specific quantification of brain amyloid (Klunk et al., 2004; Cohen and Klunk, 2014). It is currently the most widely used PET Aβ ligand for the early identification and diagnosis of AD. It can visualize the deposition of Aβ in the brain before the onset of clinical symptoms (Bateman et al., 2012). Several studies have confirmed the correlation between PET/CT measurements and histological evidence of deposited Aβ (Ikonomovic et al., 2008; Choi et al., 2012; Okazawa et al., 2020). Quantitative metrics such as the standardized uptake ratio (SUVR) and the distribution volume ratio (DVR) have been used to effectively differentiate healthy individuals from those with AD. Previous studies have shown that in patients with MCI and AD, ^11^C-PIB binding in the cerebellum is negligible and that the cerebellum is a better choice as a comparison region for PET quantification. SUVR of each brain region can be obtained by dividing the standard ^11^C-PIB uptake values in each brain region by the cerebellar uptake values (Herholz, 2003). However, the total scan and wait time for SUVR/DVR would add up to at least 1 h. Recent studies have found that the amyloid quantification index (AQI) can differentiate MCI from AD and predict the progression of MCI. AQI was calculated linearly by combining AQI_roi of the featured brain regions, and its coefficients were determined by linear regression. AQI measured the difference between PiB retention and brain tracer clearance with higher accuracy and sensitivity than SUVR and DVR (Shen et al., 2022). A combination of AQI with magnetic resonance imaging (MRI) can be used to predict the progression of MCI with higher accuracy than that with AQI alone (Patel et al., 2020). Most patients with AD’s ^11^C-PIB are positive and have an elevated cortical distribution volume ratio. Aβ imaging enables the study of the relationship between amyloid deposition and brain structure in patients with AD. Furthermore, it also aids in understanding the function of Aβ through normal aging and the changes that occur during the progression to AD. In addition, it aids in monitoring of the biological effects of anti-Aβ drugs on neurodegeneration and cognitive decline (Cohen et al., 2012). Previous studies have shown that significant differences in PIB retention are observed in regions known to contain amyloid deposits, such as the frontal and parietal cortices and the striatum (Tryputsen et al., 2015). Studies on ^11^C-PIB PET have shown strong cortical retention in almost all patients with AD (Rabinovici et al., 2007). Furthermore, ^11^C-PIB PET cortical preservation is associated with brain atrophy as measured using MRI (Chételat et al., 2010).

In MCI, ^11^C-PIB positivity is an important predictor of progression to AD, and Aβ-positive individuals are more likely to progress to AD. Presence of amyloid deposists can also predict eventual progression to MCI or AD in individuals with normal cognitive function. Therefore, it aids in early detection, early diagnosis, and interventions (Jansen et al., 2015; Brown et al., 2019). To date, the pathological relevance of large-scale ^11^C-PIB-PET/CT studies to detect MCI has been less explored. The conversion rate of MCI to AD has been reported to be between 8 and 16% per year (Mitchell and Shiri-Feshki, 2009). 50–60% of the subjects with MCI are positive for ^11^C-PiB-PET/CT, and 10 to 15% of Aβ-positive MCI eventually convert to AD each year (Rowe and Villemagne, 2011). A longitudinal study with a 3-year follow-up showed that 70% of ^11^C-PIB-positive patients with MCI eventually developed dementia (Okello et al., 2009). A systematic review that included 9 study cohorts with 300 patients with MCI found that the estimated sensitivity of ^11^C-PIB-PET/CT to identify patients with MCI who convert to AD was 96% (95% CI 87 to 99%) (Zhang et al., 2014). Ciarmiello et al. (2019) found a linear relationship between increased amyloid deposition and memory dysfunction, and proposed a SUVR threshold of 1.3 to identify MCI populations at risk of progression to AD. ^11^C-PIB uptake increases early in the progression to AD and then plateaus, with an S-shaped increase in Aβ load during the transition from normal aging to MCI and AD (Koivunen et al., 2011). Additionally, numerous studies have shown that patients with AD have significant bilateral retention of ^11^C-PIB in the precuneus, temporal lobe, superior limbic gyrus, and cingulate gyrus, as well as in the right insula and nucleus accumbens (Cohen and Klunk, 2014; He et al., 2015; Byun et al., 2017; Wenjun et al., 2017).

Furthermore, previous studies have confirmed that ^11^C-PIB-PET/CT has a sensitivity of 83.3–100% and a specificity of 41.1–100% to predict the transformation of MCI to AD (López-de-Eguileta et al., 2020). Therefore, ^11^C-PIB-PET/CT is an important neuroimaging tool for the clinical diagnosis of AD, with high sensitivity. The ability to accurately quantify Aβ protein deposition at different stages of AD provides important imaging evidence for the early identification and diagnosis of AD and provides an important basis for early anti-Aβ therapy and efficacy testing. However, the short radioactive half-life has been a major limitation of ^11^C-PiB PET, preventing its widespread use, and therefore, other ^18^F-labeled PET tracers have been developed (Byun et al., 2017). Recent studies have shown that the three ^18^F-labeled tracers are also highly consistent in terms of diagnostic accuracy, and have 89–97% sensitivity and 63–93% specificity in differentiating AD from MCI with similar results in visual and quantitative analysis (Landau et al., 2014; Prestia et al., 2015). A meta-analysis (Morris et al., 2016) found that the three ^18^F labeled tracers had better diagnostic accuracy in distinguishing patients with AD from healthy individuals. Recent studies have shown that ^18^F-flutemetamol uptake is negatively correlated with scores on neuropsychological test scales, with higher tracer uptake associated with lower scores (Heurling et al., 2016).

Previous studies have found that the sites of Aβ deposition that are significantly different between AD and MCI are the inferior frontal gyrus (left), the superior frontal gyrus (left), the parahippocampal and perirhinal gyrus (left and right), the syrinx (left and right), the postcentral gyrus (left), the cuneus (right), the nucleus accumbens (left and right) and pallidum (right) (Klunk et al., 2004; Jack et al., 2009; Koivunen et al., 2011; He et al., 2015). A recent study showed that in patients with MCI, visuospatial function is usually correlated with the degree of Aβ burden in the frontal regions, while in patients with AD, the Rey Complex Figure Test scores negatively correlated with SUVR in the frontal, temporal, parietal, occipital, anterior cingulate, posterior cingulate, and posterior cingulate gyri (Park et al., 2020). The frontal lobe is an important brain region for situational memory extraction and is closely related to memory function in humans, and the deposition of Aβ may lead to neuronal damage in the frontal areas, further leading to impaired memory function in patients with AD (Jiménez-Bonilla et al., 2019). Roh et al. (2011) showed that the basal ganglia regulate motor neural pathways in the brain and are involved in higher cognitive functions such as emotional expression and associative memory, while the nucleus accumbens is in the basal ganglia and can be altered in the early stages of AD, leading to an overall decrease in cognitive function. In addition, a recent study has shown that the cortical nucleus is involved in higher cognitive functions, such as emotional expression and memory. Furthermore, a recent study (Park et al., 2020) found significant hemispheric asymmetries in these brain regions that correlate with neuropsychological scale scores, with a higher concentration on the left side, which can be associated with major cognitive deficits in patients. In conclusion, Aβ deposition may cause neuronal cell damage in the above brain regions, leading to cognitive impairment, but further pathological and neuroanatomical exploration of the specific disease mechanism is needed.

### 3.2. Synaptic vesicle glycoprotein 2A PET/CT

Synapses are critical for cognitive function, and cognitive impairment is strongly associated with loss of synaptic density, which is observed in the early stages of clinical AD, accompanied by loss several presynaptic proteins (Robinson et al., 2014). Therefore, evaluation of synaptic density is valuable in AD research and enhancing treatment efficacy. A new approach to imaging synaptic density using synaptic vesicle protein 2A (SV2A) has recently emerged, and several SV2A ligands have been developed and translated for human use. SV2A PET can directly display synaptic density in AD. ^11^C-UCB-J is a recently developed PET tracer (Carson et al., 2022) it was demonstrated that SV2A binding was extensively reduced in the medial temporal and neocortical regions of the brain of patients with early AD (Mecca et al., 2020). Chen et al. (2021) also obtained similar results, with ^11^C-UCB-J binding showing a similar reduction in the medial temporal lobe of patients with AD compared to healthy participants. Recent studies have found that patients with AD have higher tau deposition and lower hippocampal SV2A binding (Mecca et al., 2022). O’Dell et al. (2021) measured the volumetric ratio of the ^11^C-UCB-J distribution and Aβ deposition in ^11^C-PiB and observed a significant negative correlation between overall Aβ deposition and hippocampal SV2A binding in patients with MCI. Furthermore, low MMSE scores in patients with MCI are also reported to be associated with lower SV2A binding in structures such as the hippocampus, parahippocampal gyrus, and inferior and superior temporal gyri (Vanhaute et al., 2020). Studies have shown that a deposition of Aβ in the parahippocampal and peripheral gyrus of the left hippocampal is associated with impaired cognitive function, and that the parahippocampal and peripheral gyrus of the hippocampal is an important component of the medial temporal lobe, responsible for the extraction, encoding, and highly correlated with situational memory function (Burgess et al., 2002). The sinohypothalamic gyrus is a cortex associated with the encoding of visual signals and is related to working memory processing and spatial localization. Impaired working memory processing and spatial localization in AD are also correlated with Aβ protein deposition in this region of the brain. Higher uptake of ^18^F-florbetapir is associated with a lower uptake of ^11^C-UCB-J, and both are associated with altered synaptic function in the occipital lobe (Coomans et al., 2021). However, due to their small sample size, these studies are limited in their ability to investigate the effects of important demographic variables. Future studies with larger sample sizes and greater educational attainment may help better understand the interaction of these variables with AD.

### 3.3. FDG-PET

FDG-PET is used to reflect regional glucose depletion directly related to the local intensity of brain glutamatergic synaptic and astrocytic activity (Reivich et al., 1977). Regional changes in neuronal activity caused by neurodegeneration can be sensitively reflected by regional depletion of brain glucose (Drzezga et al., 2005). The decrease in brain metabolism detected with FDG-PET is a hallmark of neurodegeneration, and FDG-PET is useful for early diagnosis, as it can reveal the characteristic pattern of AD neurodegeneration in individuals with MCI who will transition to AD earlier. Normal FDG-PET can predict clinical stability over several years of follow-up (Iaccarino et al., 2019) and abnormal FDG-PET is associated with an increased risk of progressive cognitive deterioration (Caroli et al., 2015). Several studies have shown that ^18^F-FDG PET is strongly correlated with patient symptoms and clinical severity, and it could be an ideal tool for disease staging and follow-up (Herholz, 2012). Studies have shown the added value of FDG-PET in predicting conversion to AD dementia in patients with MCI compared with conventional cerebrospinal fluid or MRI scans, especially for short-term progression (Blazhenets et al., 2020). Shaffer et al. (2013) found that combining MRI, FDG-PET and cerebrospinal fluid testing significantly improved the accuracy of predicting AD transformation compared to clinical testing alone. A recent study found that ^18^F-FDG metabolism showed a similar degree of reduction in the medial temporal lobe of patients with AD (Chen et al., 2021). Ottoy et al. (2019) found that hypometabolism of the hippocampal volume based on NMR flattening and the posterior cingulate gyrus based on ^18^F-FDG-PET was an accurate indicator of short-term conversion to AD dementia in patients with MCI at 80 and 83%, respectively, while the combination of neuropsychological tests (visuospatial construction skills), the hippocampal volume based on NMR flattening and ^18^F-FDG-PET had a specificity of 96% and sensitivity was 92%. In summary, PET/CT studies combined with other neuroimaging, blood, cerebrospinal fluid, and neuropsychological tests can provide a deeper understanding of the pathogenesis and progression of AD.

## 4. Neuropsychological test scales in Alzheimer’s disease and mild cognitive impairment

Neuropsychological tests are currently an important and cost-effective tool for diagnosing AD and MCI and differentiating subtypes of MCI (Espinosa et al., 2017). The most common neuropsychological tests used in clinical practice are the Clinical Dementia Rating (CDR), MoCA, MMSE, Activity of Daily Living Scale (ADL) (Lim and Loo, 2018). Hughes et al. (1982) developed CDR in 1982 and Morris (1993) published a revised version in 1993. The CDR provides a simple clinical assessment tool to grade the degree of cognitive impairment and activities of daily living clinically. The test takes approximately 40 min to perform and assesses six items separately—memory, orientation, judgment and problem solving, social interaction, family life and hobbies, and independent living; the severity scale is graded as healthy, suspected dementia, mild dementia, moderate dementia, and severe dementia, with scores based on a decrease in a specific area because of impaired intelligence, and no scores based on other causes such as disability (Morris, 1993). CDR values of 0.5, 1, 2, 3 are considered suspected, mild, moderate, and severe dementia, respectively (Julayanont and DeToledo, 2022). CDR has important clinical applications in differentiating dementia from MCI. MoCA (Hoops et al., 2009) is a rating scale primarily used for rapid screening of MCI (O’Driscoll and Shaikh, 2017). The cognitive domains assessed include visual space, execution, naming, memory, attention, language, abstraction, and orientation, and the scale is more comprehensive than the MMSE. Nasreddine et al. (2005) proposed a threshold of 26-point scale in 2005. They suspected patients with scores below 25 had MCI and O’Driscoll and Shaikh (2017) adjusted the score with 1 point for individuals with 12 years of education or less to account for educational effects. The MoCa test task is more complex but more sensitive than MMSE and takes approximately 10 min to detect patients with MCI and mild AD (Nasreddine et al., 2005; Hoops et al., 2009; Horton et al., 2015).

MMSE (Nieuwenhuis-Mark, 2010) is a widely used clinical cognitive screening scale that includes items for orientation, memory, recall, naming, attention, executive and visuospatial items. It has a total score of 30, with higher scores showing better cognitive function. The MMSE is a simple 5–10-min test that assesses cognitive deficits due to lesions in the left hemisphere (Jia et al., 2021). However, the MMSE is limited by the fact that the scores are influenced by age, education, and cultural background (Anderson, 2019). At older ages (>75–80 years), MMSE scores are lower and can overestimate the severity of cognitive impairment. Furthermore, clinicians should interpret MMSE results with caution in patients with very low levels of education (for example, those who cannot read), as their total MMSE score may be lower for this reason. Despite their cognitive impairment, patients with higher education may obtain a higher MMSE score (Piersma et al., 2018). The Chinese MMSE stratifies the diagnosis of AD based on educational attainment: ≤19 points for the illiterate group, ≤22 points for the elementary school group, ≤23 points for the middle and high school groups, and ≤26 points for the higher education group, with an average of ≤23 points (Jia et al., 2021). Most studies in China and abroad set the MMSE scores of patients with MCI at 25–27, and the census scores are commonly set at 24–28 (Sachdev et al., 2015). Additionally, the MMSE is a brief screening test that only superficially assesses the cognitive domains of visuospatial and executive functions (Tombaugh and McIntyre, 1992; Nieuwenhuis-Mark, 2010). The MMSE is more appropriate than the MoCA for patients with AD with more severe functional impairment (Nasreddine et al., 2005). The MMSE memory assessment is simple and shows a high sensitivity in those with severe cognitive dysfunction compared to patients with early stage of the condition and those who show only situational memory impairment. The MoCA scale is better than the MMSE in assessing various cognitive functions and evaluating memory (Pinto et al., 2019). Several previous studies have found lower MMSE and MoCa scores in AD than in MCI, suggesting that cognitive impairment is more severe in patients with AD than in patients with MCI (McKhann et al., 2011; Lim and Loo, 2018).

Other neuropsychological scales commonly used in clinical practice include the Alzheimer’s Disease Assessment Scale (ADAS-cog), the Revised Hasegawa’s Dementia Scale (HDS-R), the Memory Alteration Test (M@T) (Rami et al., 2007), and Quick Screen for Mild Cognitive Impairment (Qmci) (O’Caoimh et al., 2012). In addition, we have used the Hachinski Inchemic Score (HIS); the clock drawing test (CDT), and the global deterioration scale (GDS). A variety of cognitive tests have good diagnostic precision and MMSE, MoCA, and CDR are commonly used to assess the degree of dementia in patients. The HIS is often used to distinguish vascular dementia and mixed dementia. The M@T and Qmci tests are brief tests developed for the diagnosis of MCI and have high sensitivity (Breton et al., 2019). The Hamilton Depression Scale (HAMD) and Hamilton Anxiety Scale (HAMA) were used to identifying and exclude patients with depression and anxiety, respectively. Park et al. showed that patients with early-onset AD had poor performance on the anterior and posterior finger, visual breadth, visual counts, Rey complex map tests, suggesting poorer attention and visuospatial function and specific cognitive dysfunction in patients with AD (Park et al., 2020). Each of the scales have specific strengths and limitations; Hence, using a combination of these scales can help improve the detection rate of AD and MCI, assess the efficacy of AD treatment, and guide rehabilitation. Combining neuropsychological testing scales with clinical history, symptoms, signs, imaging, and laboratory tests is essential for comprehensive and holistic clinical assessment.

## 5. Vignette and outlook

With the aging of the population, neurodegenerative diseases, primarily Alzheimer’s disease, have become a serious health risk among older individuals and a major global health problem, affecting the quality of life of patients and imposing a heavy burden on caregivers and society. Early diagnosis and treatment can slow cognitive decline and reduce the appearance of behavioral and psychiatric symptoms associated with AD. PET/CT provides certain imaging evidence to identify MCI that may progress to AD, and in combination with neuropsychological examination, can improve the accuracy of the early diagnosis of AD and give patients the opportunity to intervene early. Few large-scale ^11^C-PIB-PET/CT studies have been conducted to explore the pathology of AD and MCI, and researchers need to further explore the correlation between PET/CT combined with neuropsychological test scales in disease progression and assessment of AD and MCI.

## Author contributions

All authors listed have made a substantial, direct, and intellectual contribution to the work and approved it for publication.

## Funding

This study was supported by Jilin Provincial Science and Technology Development Program Project (20230402012GH), Jilin Provincial Engineering Laboratory of Memory and Cognitive Disorders (2020C054), and Jilin Province Science and Technology Development Plan Project (20200404076YY).

## Conflict of interest

The authors declare that the research was conducted in the absence of any commercial or financial relationships that could be construed as a potential conflict of interest.

## Publisher’s note

All claims expressed in this article are solely those of the authors and do not necessarily represent those of their affiliated organizations, or those of the publisher, the editors and the reviewers. Any product that may be evaluated in this article, or claim that may be made by its manufacturer, is not guaranteed or endorsed by the publisher.
